# Dominant-Negative Proteins in Herpesviruses – From Assigning Gene Function to Intracellular Immunization

**DOI:** 10.3390/v1030420

**Published:** 2009-10-19

**Authors:** Hermine Mühlbach, Christian A. Mohr, Zsolt Ruzsics, Ulrich H. Koszinowski

**Affiliations:** Max-von-Pettenkofer Institut, LMU, Feodor-Lynenstr. 25, 81377 Munich, Germany; E-Mails: muehlbach@lmb.uni-muenchen.de (H.M.); mohr@lmb.uni-muenchen.de (C.A.M.); ruzsics@lmb.uni-muenchen.de (Z.R.)

**Keywords:** dominant-negative, essential genes, random mutagenesis, conditional gene expression, deletion, intracellular immunization, herpesvirus, conserved gene blocks

## Abstract

Investigating and assigning gene functions of herpesviruses is a process, which profits from consistent technical innovation. Cloning of bacterial artificial chromosomes encoding herpesvirus genomes permits nearly unlimited possibilities in the construction of genetically modified viruses. Targeted or randomized screening approaches allow rapid identification of essential viral proteins. Nevertheless, mapping of essential genes reveals only limited insight into function. The usage of dominant-negative (DN) proteins has been the tool of choice to dissect functions of proteins during the viral life cycle. DN proteins also facilitate the analysis of host-virus interactions. Finally, DNs serve as starting-point for design of new antiviral strategies.

## 1. Scope

In this article, we will highlight the possibilities of dominant-negative (DN) proteins as tools to elucidate gene functions, pathways and processes. Potential benefits of DN proteins as antiviral agents in intracellular immunization are mentioned.

## 2. The Way to Assign Herpesviral Gene Functions

### 2.1. Conservation of genes and protein function in herpesviruses

**Figure 1.**
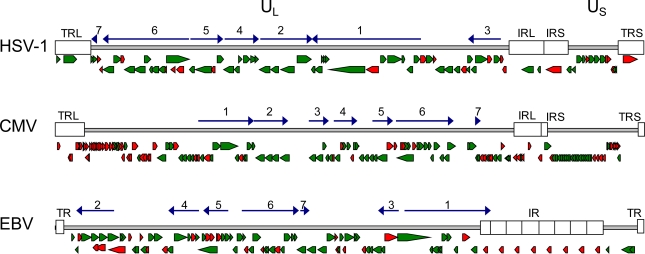
Gene maps of HSV-1, HCMV and EBV genomes. The relative orientation of the known genes for HSV-1 (a), HCMV (b) and EBV (c) are shown as arrows (modified from Fields, Virology [13]). Green arrows assign genes of which the function is known, suggested or highly predicted. Red arrows assign genes with unknown gene function [14]. Herpesviruses share a core set of 43 highly conserved genes that are organized in 7 conserved gene blocks. Here the relative orientation of the core gene blocks is depicted as dark blue arrows. In the core gene blocks most genes are essential and highly conserved among herpesviruses of different subfamilies. The major function of these genes is known or still under investigation. Most genes with yet unknown function are non conserved genes at the genome termini. (Accession numbers: HSV-1: X14112; HCMV: NC_001347; EBV: NC_007605). UL, US: unique regions; TR, TRL, TRS: terminal repeat; IR, IRL, IRS: internal repeats.

The family of herpesviruses comprises important human and many veterinary relevant pathogens. Classification of herpesviruses and separation from other virus families is predicated on virion structure, cell tropism and virus host range [1]. Their common feature is their ability to infect the host for life. There are important other reasons why herpesviruses stand out from other virus families namely their high complexity and their perfect adjustment to their host. As more and more sequence information of herpesviruses accumulate, analysis and comparison of the genomes allow the identification of core genes that are shared within different subfamilies, and unique genes that resemble adaptation to fulfill host specific prerequisites. The number of protein coding genes, also according to their genome size, vary between the subfamilies whereby α- and γ-herpesvirinae have on average around 70–80 genes and β-herpesvirinae around 160–230 [2]. There is a core set of 43 highly conserved genes, shared by all herpesviruses, and that are mainly involved in the basic and fundamental procedures of the viral life cycle as entry into the cell, DNA replication, packaging of the genome and maturation of infectious particles [3]. Although herpesviruses are extensively studied there are still genes in the list of the core genes with unknown function [4] (Figure 1). The additional species and subfamily specific genes cover areas of cellular tropism, host shut-off or anti-apoptotic processes, evasion from the immune system and maintenance of latency [5,6,7,8]. It is very likely that most of the remaining genes of unknown function will belong to one of these groups. Not all of these genes are necessary for viral replication in the host, but shape the outcome of the infection [9].

### 2.2. Strategies to identify herpesviral gene function

In order to identify a function of an unknown gene biological and biochemical assays of the wild-type (wt) protein or the deletion or mutation of the gene, as well as the use of DN mutants [10] and the study of their respective phenotypes and meanwhile also computational alignment to homologues genes of known function can be used [11,12].

#### 2.2.1. Targeted mutagenesis for genetic analysis of essential herpesviral genes

One of the most fundamental questions to address when studying a viral gene is whether the gene is essential for the viral life cycle in tissue culture or tropism for a specific cell type. At the beginning of herpesviral studies essential genes have been found by random screen of temperature-sensitive mutants [15,16,17]. Genes essential for a function in only a certain cell type *in vitro*, therefore determining tropism, have been identified by genome analysis of strains attenuated after propagation in specific cell lines [18]. Therefore, deletion or disruption of the gene of interest is an important step in evaluating its biological role. The first targeted mutations in herpesviruses have been achieved by site-directed homologous recombination of plasmids with transfected viral DNA in tissue culture [19], a procedure that is limited by the low frequency and specificity of recombination. A milestone in modifying herpesviruses was the cloning of their entire genome as bacterial artificial chromosomes (BAC), which allows the complete construction of a mutant herpesvirus genome in a controlled manner prior to the reconstitution of infectious progeny [20,21,22]. A simple, random approach to identify essential genes has been the transposon-mediated mutagenesis of herpesviral genomes encoded in BACs, whereby transposon insertion sites were mapped by direct sequencing and the viability or non-viability of virus progeny has been determined from the mutated genomes by reconstitution in tissue culture [23,21,24,25,26]. While this approach was very successful for the identification of interesting essential genes [27,28], complete analysis to identify all essential genes (at least for certain cell types *in vitro*) has to be done gene by gene [9]. Besides the deletion phenotype, expression timing (immediate-early, early and late) as well as protein localization give helpful clues to the role of a herpesvirus gene and might allow a classification to a certain step of the replication cycle, achieved by classical biochemical studies. Isolated cloned herpesviral genes can be used to study the function in non-infected cells, as for example done for the protein localization of a library of Herpes simplex virus (HSV), human cytomegalovirus (HCMV) and Epstein-Barr virus (EBV) genes shown by the group of Frappier [14]. Nevertheless, analysis of protein functions and localization in the context of the infection is indispensable, and mutation and tagging of the target gene has to be done in the viral genome. The principles of reverse genetics might offer manifold approaches to analyze mutations that abolish localization or binding to partners and, therefore, can give further hints to its function in a pathway. Introduction of the mutations can be made either in the original position of the gene using traceless mutagenesis allowing the most subtle substitutions [29,30] or in a faster way by introducing them at an ectopic side e.g. via FRT/Flp mediated recombination, which avoid off-target effects in the endogenous locus that might be responsible for the phenotype [31].

#### 2.2.2. Identification of gene function via homology screening

Computational biology provides information about different viruses and hosts and predictions to assign functions to genes can be made by sequence alignments, motif searches and structure modeling. Meanwhile 49 herpesviruses genomes are completely sequenced and stored in the GeneBank database (http://www.ncbi.nlm.nih.gov/genomes). With the recent advances of new sequencing technologies, it is obvious that sequence comparison will get even more meaningful over the years. Comparison of conserved genes of different herpesviruses can help to identify important regions and motifs, as done e.g. for pUL89 of HCMV [32]. Not only homologies to other viral genes, but also to cellular genes can be very interesting as genes might have been acquired by horizontal gene transfer, to abuse the host machinery for viral purposes [33]. Several programs allow the search for patterns and motifs that can give valuable hints in which process a gene is involved or reveal functionally relevant sequences of a protein[34], as for example PPXY motifs indicating functions in budding or DNA binding domains in DNA replication, cleavage or packaging. Yet to date there are several viral genes where no clear homology can be found and bioinformatic analysis will not provide any information about a putative biological role.

## 3. Dominant-Negative Proteins

In need of novel strategies to dissect functions of protein complexes and their roles in diverse pathways, the use of DN mutants arise to be an important and forward-looking strategy in virology. Before going into detail how DN mutants can be and have been used in the field of herpesvirus biology, the term ‘dominant-negative’ requires an explanation.

In 1987 Ira Herskowitz reported in Nature, how cloned genes altered to encode mutant products are capable to inhibit the wt gene product in a cell, thus causing the cell to be deficient in the function of the gene product [10]. In diploid eukaryotic organisms, genes are present in two alleles, one from each parent. As a consequence, two different versions of the gene product can be present in the cell. Mutants of one allele, which also inhibits the other wt allele product to fulfill its function, are called ‘dominant-negative’, as they rule over the intact protein. Therefore, in herpesviral genomes that encode for only one allele, DN genes have to be complemented either *in cis*, by an additional viral expression cassette or *in trans*, by the host cell.

### 3.1. Mechanism of DN proteins

**Figure 2.**
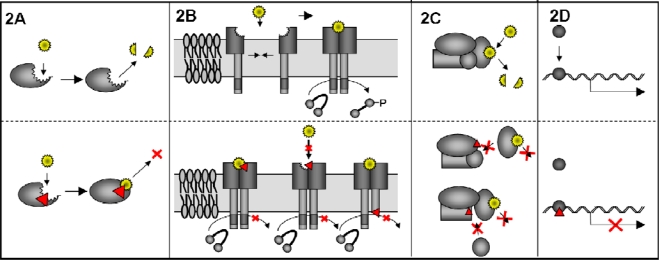
Mode of action of DN proteins. DN proteins can inhibit the function of the wt protein in different ways. A) Mutation (red arrowhead) in the catalytic domain of an enzyme may lead to binding of the substrate (yellow star) but no conversion. B) Schematic view of a phosphorylation reaction that is dependant on substrate binding and homo-dimerization of the DN membrane protein. Mutation of the substrate binding site may lead to a bound substrate, that is not released anymore or the binding of the substrate itself is inhibited. Mutation in the active site of one of the homo-dimers will not allow reaction on the target molecule. In all cases phosphorylation of the target molecule is impaired. C) The function of a multi subunit complex can be influenced by mutation of different subunits of the complex. In all cases binding of the DN subunit competes with binding of the wt subunit. Here the DN protein does not allow binding of another necessary subunit that is needed for the whole multi-complex function. D) DN mutation in a transcription factor blocks the binding site for the wt transcription factor and thereby inhibiting the downstream gene expression.

There are different methods how a DN mutant may work, which are reviewed in detail from Veitia [35,36]. Mutations in the catalytic site of an enzyme are one way how a DN mutant can arise; in that case the substrate is bound but not converted and so the balance of the reaction is disturbed. Examples in cell biology are mutations in ATPases or GTPases [37,38,39], especially Rho GTPases have been widely used [40] (Figure 2A). Very often DN proteins work in complexes, where the inhibitory potential ranges from the block of a simple dimer, as for example membrane receptors or transcription activators, which can transmit a signal only after dimerization [41,42] (Figure 2B), up to multi-subunit complexes (Figure 2C), demonstrated nicely by Barren and Artemyev for G-protein alpha subunit complexes [43]. DN mutations in transcription factors can poison a whole pathway if they block the binding sites for active mutants, therefore, inhibiting all downstream gene expression. As for example in the case of DN mutants of the proto-oncogene p53, resulting in the loss of growth inhibition and cancer manifestation [44] (Figure 2D).

The example of a DN mutant that acts in the wt form as a homo-dimer explains why a DN mutation can cause a stronger phenotype than a deletion (in case of a diploid eukaryotic organism). By equal expression of wt and DN, homo-dimerization of the two proteins will lead to the formation of wt-wt, DN-wt, wt-DN and DN-DN complexes. Therefore only 25% of the dimeric complexes will be functional, while in a deletion it would be 50%. Overexpression of the DN protein shifts the ratio more to non-functional complexes. As (random) insertion into the eukaryotic genome is still easier to achieve than targeted deletion of both alleles, it is obvious why the DN approach is superior to deletion mutants. The problem of targeted deletion was partially overcome by the RNAi approach [45], where complementary siRNA molecules initiate the degradation of the mRNA of both alleles. A drawback of this method is still that down regulation is rarely complete and the knock down is dependent on the half life of the gene product.

Furthermore, DN proteins have one big advantage that cannot be substituted by any deletion, namely that they can arrest the complexes or pathways at different steps. Often proteins are dynamic and have several functions as they bind to many other proteins or have different localizations depending on activation status. Mutating one domain to make it a DN protein, can lead to the disturbance of only one function while leaving other domains intact. Therefore, not the most prominent phenotype due to the loss of the protein, which may in fact reflect the sum of several functions, but several arresting steps can be monitored.

**Figure 3.**
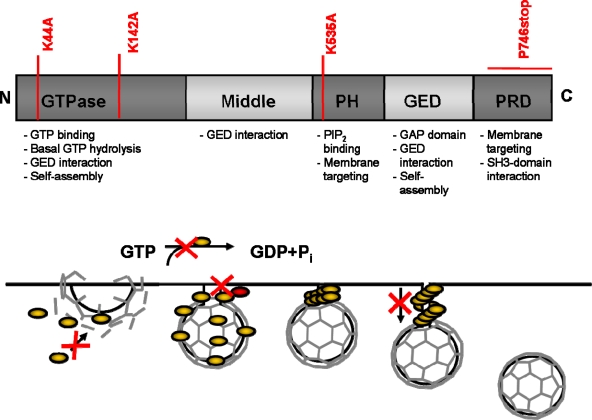
Dynamin – a proteins function explained by DN mutants. A) The Dynamin protein consists of five different functional domains. Mutations of Dynamin (marked in red), at different positions of the protein, generated DN mutants that inhibit different wt functions of Dynamin. The different functions of the domains are summarized below the scheme. PH: pleckstrin homology domain; GED: GTPase effector domain; PRD: proline-rich domain. B) Schematical overview of the function of Dynamin in endocytosis. Dynamin is recruited to clathrin-coated pits and via GTP hydrolysis results in conformational change before, or concomitant with, vesicle scission. Marked in red are the different steps where DN mutants of Dynamin could block and were used to investigate Dynamin wt functions.

An example for this postulate is the cellular protein Dynamin (Figure 3) that is involved in clathrin-coated vesicle endocytosis. It consists of five domains, a N-terminal GTP hydrolysis domain, a middle domain, a pleckstrin homology (PH) domain a GTPase effector domain (GED) and a C-terminal proline-rich domain (PRD) [46]. A well studied DN mutant is the DynaminK44A, a mutant that cannot bind GTP resulting in a block of receptor-mediated endocytosis [47,48]. Mutation of residue K535 in the PH domain, also giving rise to a DN phenotype, acts by co-oligomerizing with endogenous wt Dynamin and indirectly impairs phosphoinositide binding [49,50]. Deletion of the PRD domain by a stop codon in place of the Proline 746 of Dynamin interferes with the recruitment of Dynamin to clathrin–coated pits and inhibits in a DN fashion receptor-mediated endocytosis [51]. Together with the finding that another mutation in the GTPase domain K142A is defective in its ability to change conformation although still hydrolyzing GTP, the hypothesis was postulated that the function of Dynamin in endocytosis requires both GTP hydrolysis and a resulting conformational change before or concomitant with, vesicle scission [52]. Thus, the use of different DN mutants of the same protein can reveal more information than a deletion could have given, as they represent states that can be perceived as snap shots of highly dynamic processes.

## 4. Elucidating Herpesvirus Biology with the Help of DN Proteins

Mutants of cellular proteins such as Dynamin can resolve their functions. Of course DN mutants can also be used as tools, by inhibiting known steps in cellular pathways and, thereby, analyzing the effects on viral infection. In case of Dynamin, DN mutants of the protein serve to study whether viruses use receptor-mediated clathrin dependent endocytosis as entry pathway. Thereby, the necessity of Dynamin for entry of HIV-1 could be shown [53], but also the fact that HPV-16 does not need this entry pathway [54].

### 4.1. Identification of pathways with cellular DN proteins

For herpesviruses entry via receptor-mediated endocytosis or via receptor-mediated fusion has been postulated [55]. Evidences for both cases exist and might depend on the cell line used for the study. Interestingly, use of DN versions of the focal adhesion kinase (FAK), Src-Kinases and RhoGTPase, resulted in decreased uptake of Kaposi’s sarcoma associated herpesvirus. This led to the hypothesis, that upon binding of the virus to surface receptors, signaling of integrins to FAK activates Src. This Src activation may then recruit Clathrin and Dynamin to the cell surface, allowing the bound virus to enter the cell via newly formed vesicles [56,57,58,59]. Although viral entry mechanisms are still a matter of debate, usage of defined DN mutants can help to elucidate the complex process of subsequent steps that happen at the cell membrane.

After entry, capsids are transported to nuclear pores and the viral DNA is released into the nucleus, where viral DNA replication takes place. Study of the cell cycle arrest induced by herpesvirus and the resulting apoptosis has been profiting from the huge set of available DN cyclins. Abusing their original function in cells, herpesviruses bind to cellular cyclins to control late gene expression. Advani and colleagues could demonstrate that a subset of γ2-proteins are not produced in cell lines expressing DN cdc2 [60]. Usage of cyclin D3^DN^ helped to understand the localization and function of ICP0 of HSV [61]. These examples are just illustrations of what can be or has been done with the help of cellular DN proteins.

Recently, we could prove the anti-apoptotic feature of M36 of murine cytomegalovirus (MCMV) by replacing the viral gene with a DN FAS-associated via death domain (FADD^DN^). While the deletion virus ΔM36-MCMV was severely impaired, inhibition of the apoptosis pathway by FADD^DN^ rescued virus replication *in vitro* and *in vivo*. This novel approach of inserting cellular DN proteins into the virus genome has enabled us to define the biological function of M36 and might represent a strategy to evaluate other anti-apoptotic viral genes [62].

### 4.2. Identification of pathways with viral DN proteins

Viral DN genes are in wider use than cellular DNs [63,64,65,66,67,68,69] to analyze viral replication cycles. Instead of giving further examples of what can be done with viral DNs in terms of identifying pathways and processes on the cell level, we will address the question what can be done in the living organism.

The protein UL9 of HSV1 is the origin binding protein and, therefore, essential for viral replication [70]. After successful inhibition of HSV-1 infection in cell culture by the DN protein UL9-C535C (UL9^DN^) Yao and colleagues created a strongly attenuated recombinant herpesvirus (HSV-UL9^DN^) that can conditionally express the UL9^DN^ [71]. The induced expression of the UL9^DN^ abolishes the expression of γ_2_-proteins almost completely as they are dependent on viral DNA replication. Co-infection of HSV-UL9^DN^ together with wt HSV-1 or HSV-2 inhibited replication of both viruses [72]. Intraocular infection of mice with HSV-UL9^DN^ did not lead to an acute herpetic infection. After challenge with the wt strain no keratitis and encephalitis was seen, due to strong neutralizing antibody titers and cell mediated immunity induced by the mutant [73]. To further increase the safety and immunogenicity of HSV-UL9^DN^ as a vaccine the gene of UL9 was deleted and an additional copy of a highly immunogenic glycoprotein (gD) inserted. Herpetic skin disease could be prevented using this new derivate CJ9-gD in the guinea pig model [74]. Although the DN approach in vaccine development must be carefully considered, as the addition of inhibitory proteins alone may not fulfill the same safety criteria as the deletion of essential genes. Furthermore it provides a new feature to vaccines as they can now also inhibit herpesvirus DNA replication of the wt type virus in the rare case of a co-infection of the same cell at the time point of vaccination.

## 5. Design of DN Proteins

In summary, utilization of viral or cellular DNs depicts a promising strategy to investigate and dissect the roles of proteins in the viral life cycle. But how can DNs be isolated? Most viral DN mutations were generated at random or found by chance. Many cellular DN mutants have been discovered by resolving the genotypes to certain inherited diseases, as for example mutations in the genes for Collagen causing Osteogenesis Imperfecta [75] or mutations in ras or p53 found in various cancer cells [44]. Often truncations of oligomeric proteins have been found to cause a DN effect [76,77], although this is not necessarily the case. The attempt to tag the small capsid protein of β-herpesviruses with the green fluorescent protein, originally with the purpose to study virus entry with a labeled capsid, generated strong DNs (GFP-SCP) [78]. In another study a library of random fragmented DNA sequences was used to identify truncated proteins that could inhibit bacteriophage lysogenicity; from 80.000 mutants only four DN proteins could be identified and the approach seems to be only feasible for this special application [79]. Random multiple point mutations, as generated by *random mutagenesis* PCR [80] do not provide direct evidence, which of the changes in the sequence is responsible for the resulting phenotype. Rational protein design could be optimal. An absolute prerequisite in that case is comprehensive knowledge and information about structural or functional domains. This information is limited to only rare cases. A systematic genome-wide screen for DN function has been applied to poliovirus. Mutations have been designed either corresponding to previously characterized lethal mutations or mutations that have been predicted by a computer algorithm to destabilize the protein structures. DN mutations were identified by co-transfecting wt and mutated genomes [81]. While this strategy might be suitable for RNA viruses with small genome size and where substantial information is already known, it is obvious that such an approach is unfeasible at present for the large genomes of herpesviruses. Therefore, to investigate a protein of choice of yet unknown function(s), random mutagenesis is a suitable starting point of investigation.

For this purpose, two methods are generally applicable, alanine-scanning mutagenesis [82,83,84] and linker-scanning transposon mutagenesis [85]. While in the alanine-scanning mutagenesis only amino acid substitutions to alanine are possible, the linker-scanning mutagenesis can not only create additional amino acid insertions but also C-terminal deletions. The production of mutant libraries with both methods is simplified by the availability of commercial products. The limiting and time consuming step is the evaluation of the received mutants.

Generation of DN herpesviral genes by transposon-based mutagenesis can be divided into three steps. In the first round, the cloned gene is mutated by a linker-scanning transposon mutagenesis approach, generating a library of mutants of the gene of interest. In the second step, mutants are reinserted into the herpesviral genome lacking the gene of interest in order to test for their ability to complement the deletion phenotype. This will provide information about essential and important regions of the gene. Then mutants that could not rescue the deletion phenotype are screened for their DN potential by inserting them into a wt herpesvirus genome in the third step.

In the following two sections, we will describe two examples in which our group successfully used linker-scanning transposon mutagenesis to identify DN mutants of M50 and M53 of MCMV.

### 5.1. Comprehensive genetic analysis of herpesviral gene functions by a random transposon mutagenesis approach

We successfully used a Tn7-transposon-based mutagenesis screen on isolated viral genes (reviewed in [86]), which introduces a 5 amino acid insertion or a stop codon and reinserted the mutated genes in an ectopic position. This random screening approach proved itself to be generally applicable and subtle enough to reduce the risk of protein misfolding. As the screen is genome based, the mutants can be studied in the biological context and is in general still suitable for high-throughput analysis. These comprehensive screens allowed us to identify important sites for functionality of the protein in terms of viability of the viral progeny, localization of the protein in the cell as well as binding sites for interaction partners. In case of the nuclear egress protein M50 of MCMV the screen revealed the importance of the N-terminal part of the protein, identified the binding site to M53 therein, the crucial necessity of the transmembrane domain and the importance of a conserved proline stretch, as well as an non-essential regions which could be used to tag the protein [87].

The next protein that we addressed with this comprehensive mutagenesis was M53, the binding partner of M50 both forming together the nuclear egress complex (NEC). Detailed analysis of the resulting mutants revealed that the very N-terminal part comprises a nuclear localization signal (NLS) that could be replaced by the NLS of SV40, the binding site to M50 could be narrowed down to a region of 31 amino acids and that there are two important domains (conserved domain 2 and 4) with other functions essential for morphogenesis of MCMV [88]. Further improvements to the transposon-based mutagenesis allowed us to cover targeted genes with insertions of on average at least every fifth amino acid (data not shown).

### 5.2. Identification of gene function by DN viral genes gained from screening of transposon libraries

As DN mutations can arrest dynamic processes at different stages and therefore allow an analysis of phenotypes that cannot be resolved with deletion mutants, we were keen to find also DN mutants in the comprehensive transposon libraries of M50. In the first screening step we used a total of 104 mutants of M50 that were inserted into MCMV-ΔM50 genomes to complement the missing gene. Thereby we found 34 mutations that possessed a loss-of-function phenotype, meaning that no viruses could be reconstituted from the transfected BAC DNA. To analyze if these mutations could also be DN, the individual mutants were inserted at an ectopic position into the wt genome. Furthermore the gene expression of DN candidates was controlled via a Doxycyclin-inducible expression cassette, which also allowed a quantification of the inhibitory effect [89]. Interestingly, no mutation that destroyed the binding site to M53 possessed a DN effect. Whereas two insertion mutants, (M50^i40^, M50^i125^) as well as the targeted deletion mutant M50^ΔVR^ were able to inhibit M50^wt^ function after induction of gene expression and therefore leading to a reduction of virus production [90]. Semi-quantitative expression analysis by Western Blotting revealed that the DN effect was dependent on the protein amount. Further immunofluorescence analysis of the NEC complex with the DN mutants showed that there was neither mislocalization nor decreased stability of the proteins. Therefore, we concluded that the DN function affected the interaction of the NEC with another yet unidentified important protein partner. Based on sequence alignment with other homologues of M50 a motif within the deleted region of ΔVR was identified and the targeted deletion resulted in an even stronger DN effect. Electron Microscopy analysis of this mutant M50^ΔP^ showed the expected phenotype, as packaged capsids accumulated in the nucleus. Furthermore, the block of capsid export was accompanied by the accumulation of membrane stacks within the nucleus.

Confirmed by the positive results from the M50 screen, we next applied the DN screen to its binding partner M53. To this end we tested 46 mutations, which could not rescue the virus MCMV-ΔM53 phenotype. Unlike the M50 screen here we could find numerous DN mutants, seven insertion mutations and four stop-mutations mainly located in the conserved region 4 (CR4) and two DNs in CR3. Furthermore, seven insertion mutations in CR2 were heavily attenuated (Popa et al., manuscript in preparation). In general, all mutations in CR4 possessed a stronger inhibitory effect than mutations in CR2. All CR4 mutants blocked nuclear egress of viral capsids by interfering with the NEC, by mislocalization or destabilization. One stop mutant M53^S309^ was very effective and could block virus egress completely. Surprisingly, this effect was not depended on binding to the NEC (in contrast to M50^DN^s) and rather revealed a new function for the M53 protein in DNA cleavage or packaging, as imperfect cleavage of unit length genomes could be observed, which has also been shown for the positional homolog BFLF2 in EBV[91]. This leads to an accumulation of immature capsids that did not contain viral genomes. Another DN mutant M53^i207^ did not influence capsid maturation, therefore indicating that the block might be happening to a latter stage in morphogenesis. These findings suggested new functions for M53.

This screen once more proves that DN proteins can help to elucidate herpesviral morphogenesis steps, beyond the analysis of gene deletion mutants by freezing processes at different stages.

While linker-scanning transposon mutagenesis to identify DN mutants has been applied to cellular genes [92,93,94], the impact of this tool has not yet drawn full attention of the herpesvirus field. While several groups already use linker-scanning mutagenesis to identify important regions in the gene of interest, they did not screen the libraries for DN mutations [95,96]. But, Lin and Spear nicely showed in 2007 that several mutations in gB, derived from linker-scanning mutagenesis, exhibit DN features; and effect expression at the cell surface and entry activity [97]. Altogether comprehensive transposon-based screening for DN proteins can be applied to any essential viral gene to identify DN mutations. Analysis of nonessential genes might be also possible depending on the availability of read-out systems for the resulting phenotype.

## 6. Intracellular Immunization - Using the Antiviral Properties of DN Proteins

Ignoring their possibilities of biotechnological implementation, DN proteins have a bad repute, as they are often accompanied by genetic diseases. But the cell can also benefit from DN proteins. The replication cycle of retroviruses needs their integration into the host genome. Therefore, most mammal genomes contain inserted retroviral sequences (up to 8% for humans [98]). On the search for natural inhibitors of retroviral replication, truncated viral proteins, artifacts from incorrectly inserted genomes where found that provide resistance against a new retroviral infection. In mouse, the friend-virus-susceptibility-1 gene for example counteracts against leukemia virus infection, inhibiting genome integration and formation of the provirus. Detailed analysis of the gene revealed its origin as a gag derivative from an endogenous retrovirus [99]. Also, in sheep mutated retroviral elements can be found that block retroviral morphogenesis [100,101]. Therefore, evolution favored the maintenance of these DN viral factors [102].

**Figure 4.**
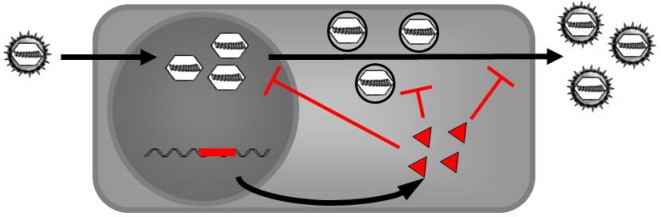
Intracellular immunization by expression of DN mutants. Stably transformed cells can express DN mutants of viral genes in trans. The DN mutant may inhibit and outcompete the wt function of the viral protein. This should inhibit viral replication. The target protein must fulfill an essential function during the viral life cycle and so DN can attack viral replication, nuclear morphogenesis, capsid assembly and packaging of viral DNA or viral exit.

Baltimore defined the term ‘intracellular immunization’ in 1988 [102], based on the finding of Friedmann and colleagues that a truncated transcription activator VP16 of HSV-1 inserted into the cellular genome can provide DN resistance against HSV-1 infection [76] (Figure 4). Therefore, Baltimore suggested this principle to be generally applicable generating antiviral resistance. There are several prerequisites to be fulfilled: First, there is the need of a strong DN effect as a potent inhibitor of viral replication. This inhibitory effect should be virus-specific without toxicity due to expression Since Baltimore’s first proposal several groups tried to fulfill this task. Especially in the field of HIV mutants of *gag*, *env* or *rev* were generated that have DN effects with the intend to produce HIV resistant T cells [103,104]. The targeted virus needs not to be a retrovirus, since a truncated N gene of rabies virus for example could inhibit viral replication in cell culture [105].

Most herpesviruses cause relatively mild syndromes in immune competent hosts. Yet, immune deficiency and in particular congenital infection is frequently associated with abortion or severe malformation. Herpesviruses also threaten lifestock animals. This ranges from equine herpesviruses (EHV) infection in horse, bovine herpesvirus (BoHV) in cow, gallid herpesvirus-2 (MDV) in chicken, pseudorabies virus (PRV) in swine also to the relatively new discovered Koi-herpesvirus (Cy-HV) which gains importance in aquatic cultures. In the USA three billion dollars per year are estimated to be lost due to BoHV-1 induced bovine respiratory disease complex [106]. For only few herpesviruses vaccines are available. Still vaccine breaks have occurred regularly, especially in case of MDV as the available vaccines only weaken the symptoms, but infected chickens can still shed virus. This can lead to the evolution of more virulent strains, which is considered as a threat [107,108]. Intracellular immunization might offer an attractive alternative to vaccination in lifestock.

Smith and Deluca produced the first mouse carrying a DN transgene with the purpose of intracellular immunization against HSV-1. Reduction of virus titer was found in 3 of 4 mouse lines. Still mice were suffering from weight loss, which was up to 60% compared to control mice, indicating toxicity of the constitutively expressed ICP4 mutant [109]. As ICP4 operates by forming a complex with the TATA-binding protein (TBP) and TFIIB to activate or repress transcription, it is possible that the DN protein still has some intrinsic potential to interact with cellular proteins and thereby disturbing transcription in the transgenic mice. Ono and colleagues could transfer the principle of intracellular immunization to PRV by a DN chimerical protein, consisting of a DNA binding domain of IE180 of PRV and a tail-truncated VP16 of HSV-1, lacking the transcription activation domain. After successful application *in vitro* [110], inhibition of viral replication was additionally shown in transgenic mice [111]. While almost all transgenic mice survived PRV challenge, proving the principle success of the intracellular immunization, the mice appeared with severe growth defects as described before in the HSV-1 resistant mice by Smith and Deluca.

Thus, side effects of constitutive DN expression are major drawbacks in the generation of virus-resistant animals. To overcome this problem an inducible expression system of DN proteins was suggested. To this end Ono’s group used a Tetracycline inducible expression system that was able to interfere with PRV replication after Doxycyclin induction *in vitro* [112]. This must be seen as a proof-of-principle, but general application in lifestock is not feasible at this stage. Therefore, we propose that a suitable expression system for intracellular immunization must be under control of the target virus. Virus responsive elements that are tightly controlled are difficult to produce, but nevertheless this seems to be a forward looking strategy.

## 7. Conclusions and Perspectives

Besides ongoing research in the herpesviral field and intense studies on genes with unknown functions, the role of the corresponding proteins are still elusive. One major problem of some proteins is that they participate in multiple processes, making studies of their function difficult. Deletion of a gene will result in a complex phenotype which is hard to explore. Here, DN mutants might be invaluable tools to dissect the function of multifunctional proteins. As described above, DNs can poison a whole complex and arrest it at a certain stage, but might allow all other functions of the protein. This could help to resolve highly dynamic processes.

Availability, of an appropriate DN is of course fundamental for this kind of studies. In this manuscript, we mentioned several possibilities to create DNs. Furthermore, we showed that a linker-scanning transposon mutagenesis is an attractive method to generate sets of DN proteins which in the case of the proteins M50 and M53 of MCMV helped us to elucidate part of their function. Due to the progresses in herpesvirus genetics, manipulation of the herpesvirus genome encoded in BACs and so providing DNs in cis is a simple procedure.

Trans-complementation of herpesviral DNs, termed intracellular immunization, is a versatile and promising experimental strategy which deserves further study. This concept proposes that expression of a DN protein could inhibit viral replication to generate viral resistance within a cell or a host. Within this manuscript we reviewed the first and important steps of intracellular immunization within the herpesviral field. The DNs in these proof-of-principle experiments are still associated with negative effects on the host. Therefore, further refinements are needed in order to make the procedure be generally applicable for creating herpesviral resistant lifestock. A virus inducible expression system, which provides the DN only in infected cells and at the time point of infection, may represent the solution.
